# The Week's Work

**Published:** 1910-06-11

**Authors:** 


### The Week's Work.

### SPECIAL DEPARTMENTS IN GENERAL HOSPITALS.

The subject of special departments in general hospitals was very exhaustively discussed before the Lords Committee some years ago, and it is still instructive to read the evidence of the various witnesses who appeared before the Committee and gave evidence in regard to this question. It is at once seen that the witnesses who were connected, directly or indirectly, with special hospitals, either deprecated or very strongly condemned the establishment of special departments in general hospitals, while on the other hand, those who were connected with general hospitals favoured the creation of "special departments as adjuncts in a general hospital." The arguments for and against the view that special departments are necessary in a general hospital mainly amount to this, that such departments are usually starved, are generally inefficient, and can never take the place of properly equipped and staffed special institutions. On the other hand, it was alleged that in all large general hospitals, especially such as possess medical schools attached to the hospital, special departments are necessary and desirable. This argument, it is true, was not laid stress upon before the Lords Committee, and probably nowadays few of those in favour of such departments would make use of it, since it is now generally held that the school exists for the hospital, not the hospital for the school, although in every case the two ought to assist one another. The great argument in favour of such special departments is that in the interests of the patients they are imperatively necessary. The time has gone by when the general surgeon was able to treat all cases that entered the surgical side of the institution, or the physician all those that came to the medical wards or to the medical out-patient department. Certain specialities have been recognised from very early times, although in England hospital specialism has not reached the stage that it has attained on the Continent, where a large hospital possesses departments for nearly every speciality. With us skin diseases, diseases of the nose and throat, and more recently of the ear, have been acknowledged deserving of special attention, and have had departments assigned to them under the charge of specialists. It is quite true that the accusation has often been made that these departments are badly carried on, ill-equipped, and under-staffed. From the point of view of a specialist and a special hospital, they are undoubtedly very often treated in a stepmotherly manner, but the fact remains that they are capable of doing good work, and that they are of incalculable benefit to many hospital patients.

### THE GENITO-URINARY DEPARTMENT AT GUY'S.

In the circumstances, the latest development in hospital specialisation in London—the creation of a special department for genito-urinary diseases at Guy's Hospital—will be watched with interest. Every large continental hospital possesses a special genito-urinary ward, and if there is one department which should be vigorously developed and well looked after in a general hospital it is this. Some specialities—for instance, orthopædics—demand so much space and so much money that the creation of special departments for them by a general hospital is hardly justifiable, and it is generally found that such departments do not take the place of special institutions, and that they only benefit a small percentage of the patients who attend them. The case for genito-urinary diseases is different. The genito-urinary department does not demand a large amount of space or special buildings—although it is desirable that all special departments should have their own exclusive domain, each, if possible, separate from the others—while its equipment and upkeep do not entail a large annual expenditure. The self-evident difficulty in the case of such a department lies in the overlapping that must necessarily occur between it and the general wards. This can only be dealt with by combined action on the part of the staff. Guy's Hospital has recently established several new departments, of which the genito-urinary promises to be the most interesting. It has been placed in charge of Mr. A. R. Thompson, a former Hunterian professor, who is at present engaged in genito-urinary work at the Hospital Neckar. Mr. Thompson is a distinguished Leeds and Guy's student, and under his charge the new department may be trusted to flourish.

### THE WEIR CHARITY.

Judgment was given on Tuesday in the Court of Appeal by the Master of the Rolls, Lord Justice Farwell, and Lord Justice Kennedy in the appeal brought by the Wandsworth Borough Council against the decision of Mr. Justice Eve in the case of the Weir Charity. The outstanding facts of the case are sufficiently well known to our readers to make their enumeration here unnecessary. The Master of the Rolls, in delivering judgment, stated that in general the doctrine of *cy-près* is limited to old charities, and that modern cases rarely occur in which the doctrine can be properly applied. There could, in this case, be no question of *cy-près* until it was clearly established that the directions of the testator could not be carried into effect. In this case the construction of the will did not present any difficulty. It was obviously the testator's intention that a hospital should be erected to be called the "Weir Hospital," and there was no evidence to show that a cottage hospital could not be established in that neighbourhood on such a scale as would absorb all the available income. It was true that a hundred thousand pounds sterling would not provide for a general hospital, but the testator did not specifically mention a general hospital, so that his directions might be taken to allow of the erection of a cottage hospital. In the circumstances there was no justification for invoking the doctrine of *cy-près*. There could be no attempt to justify the payment by the Charity Commissioners of the £5,000 to the Bolingbroke Hospital without any special scheme or without any notice of their intention to make such a payment. That money must now be applied under a special scheme so framed as to give effect to the directions contained in the testator's will. The appeal was allowed with costs. Lord Justice Farwell and Lord Justice Kennedy concurred, and the former expressed the opinion that it was alarming to find that a Government office was capable of such misapplication of funds entrusted to its care. The Charity Commissioners had no power whatever to make such a grant.

### NONCONFORMITY AND MEDICINE.

It is a commonplace observation how very largely temperament dictates opinion, and how, under its influence, unphilosophic people will reason aside contradictory facts like any Hegelian. It would be supererogatory here to dwell upon this familiar subject: all that is intended is to point out a neglected illustration of it which the medical observer, or, indeed, any observer, may verify repeatedly. The particular temperament in question is what, for want of a better phrase, may be called pro-minority feeling—the well-known preference of Cato. And the opinion to which it so surely gives rise is strong prepossession in favour of the irregular practitioner of medicine, coupled with prejudice towards the State-approved one. The most obvious argument to advance in support of this is to instance persons who by nature are revolutionists, who, sometimes rightly, sometimes wrongly, decry tradition and orthodoxy and accepted ideas. A contemporary example which suggests itself at once is Mr. Bernard Shaw. Mr. Shaw, as almost every newspaper reader knows, is a Socialist, an agnostic, a feminist, teetotaler, non-smoker, and vegetarian, who objects to punishment of crime and the wearing of mourning. No very profound student of character is required to deduce from this that he is also an anti-vivisectionist. The combination is usual. But sympathy with a minority—as a party getting the worst of things—is, of course, a central idea also in several religions of sentiment, especially in Protestant Christianity, which is inspired by the Lutheran rejection of external authority; and, above all, in that division of Protestant Christianity known as Nonconformity, which goes furthest in the direction named. We have no wish to say anything to wound Nonconformists, whose activities in some ways are wholly for good; but it is unfortunately true that before now—as in the case of Mr. W. T. Stead, with his campaign in favour of the now forgotten Mattei cancer cure—they have hindered the cause of humanity sadly by their preference for anybody who opposes recognised medical teaching. It all almost points to an essential love of the second rate, about the only fault of this body which Swift missed in his savage "Tale of a Tub."

### THE USEFULNESS OF SANATORIA.

With not the least surprise we noticed a column advertisement of the cure of consumption by E. W. Alabone, in a recent number of the *Christian World*. What attracted attention was a gross misstatement in the first paragraph, to the effect that consumptive patients are pretty unanimous in condemning sanatorium treatment, not only as a failure, but as a positive danger. In view of the rapid increase —now, after ten years' trial—in the number of these institutions, of the action of German insurance companies in providing them as a matter of business, and of the favourable report on them, after a painstaking survey, by an official of the Local Government Board, it hardly seems necessary to rebut this amazing assertion. But still it may as well be said there is not, in fact, the slightest foundation for it. A great deal more might be added, but perhaps the above will suffice, especially as elsewhere in the same issue of the journal in question there appear commendatory reviews of a book on sanatorium treatment by a medical superintendent of one of these institutions, and of Dr. Latham's and Mr. Garland's "Conquest of Consumption," which advocates the establishment of 30,000 sanatorium beds. The editorial part of the paper reflects, in fact, some of the intellectual expansion of these latter days, an expansion which Nonconformists have not failed to participate in. But why let the advertisement columns suggest ignorant or malicious prejudice?

### THE DRUG MARKET.

Several important changes in the values of drugs have taken place since the last report went to press, but business continues somewhat quiet. Following on the advance in the price of bismuth, makers of bismuth carbonate, bismuth subnitrate, and the other salts of bismuth have raised their prices by one shilling, or a little over, per lb. The advance in the price of the metal was unexpected. Buchu leaves are again substantially dearer, and the available supplies are extremely small, with little likelihood of any large quantity coming on to the market for some months to come. Coca leaves are a trifle dearer. Extreme prices have again been paid for asafetida of fine quality. Jalap still tends dearer. Oil of cubebs is dearer, owing to the continued scarcity of berries. Rhubarb is tending somewhat lower in price, as also are ipecacuanha and myrrh. The cod fishing in Norway is nearly over, and although the catch of cod is not remarkably less than last season, the yield of oil is very considerably below last season's yield. At the end of May the number of cod fish which had been caught was 50½ millions, against 52½ millions at the same date last year; but whereas the yield in oil up to May 30, 1909, was 40,700 barrels, the yield up to the same date this year was 32,700 barrels. The present price of cod-liver oil is materially below that quoted earlier in the season, but it is not improbable that the price may still recede slightly.

